# Development and validation of the Workplace Relational Needs Satisfaction Scale (W-RNSS)

**DOI:** 10.3389/fpsyg.2024.1419765

**Published:** 2024-06-27

**Authors:** Veronika Hanc, Gregor Žvelc, Boštjan Bajec

**Affiliations:** Department of Psychology, Faculty of Arts, University of Ljubljana, Ljubljana, Slovenia

**Keywords:** relational needs, personnel, interpersonal relationships, work environment, Relational Needs Satisfaction Scale

## Abstract

While inadequate relationships in the workplace pose a significant psychosocial risk, quality interpersonal relationships can contribute to positive effects and prevent negative outcomes. Erskine’s model of relational needs, not yet studied in the work environment, can provide a more detailed understanding of the needs employees experience in their workplace relationships. We adapted the general Relational Needs Satisfaction Scale (RNSS) for coworker relationships and examined the factor structure of relational needs in the workplace and their connections to various work aspects. The sample comprised 273 participants, including both employees and students, in a workplace setting with coworkers. The results show that the Workplace Relational Needs Satisfaction Scale (W-RNSS) is a valid and reliable instrument (*α* = 0.93 for the total scale and 0.77 < *α* < 0.89 for the subscales) for measuring relational needs in coworker relationships. A bi-factor model was the most suitable for describing the data (*χ*^2^/*df* = 1.94, CFI = 0.95, TLI = 0.94, NNFI = 0.94, RMSEA = 0.06, SRMR = 0.04, AIC = 13289.27, BIC = 13506.29), confirming the previously supported 5-factor structure and the general factor. Satisfaction of relational needs was associated with higher work satisfaction, increased work engagement, greater motivation and lower burnout, underscoring the importance of quality interpersonal relationships among employees. W-RNSS shows potential for researching connections with other work aspects and practical applications in prevention and intervention strategies.

## Introduction

Erskine (1998), Erskine et al. (1999), Erskine (2015), developed a model of relational needs rooted in attachment theory, object relations theory, transactional analysis, and self-psychology. These theories highlight the importance of relationships as a primary human motivation (Fairbairn, 1954; Berne, 1961; Bowlby, 1969; Kohut, 1971, 1977, 1984; Ainsworth et al., 1978; Stern, 1985; Fairbairn, 1986/1941; Winnicot, 1986/1960; Guntrip, 1992/1968). Erskine (2015, p. 46) defines relational needs as those “unique to personal contact” which can only be satisfied within a responsive human relationship. According to Erskine et al. (1999), relational needs are dynamic; with specific needs becoming prominent as longings or desires while others recede into the background. They suggest that an attuned and involved response from another person can satisfy these needs, whereas their non-fulfillment may lead to frustration and anger. Over time, persistent non-satisfaction can culminate in a loss of hope and meaning alongside negative beliefs about oneself, others, and life (Erskine and Moursund, 1988).

Erskine et al. (1999) identified eight primary relational needs frequently expressed by clients in psychotherapy: (1) for security; (2) to feel validated, affirmed, and significant within a relationship; (3) for acceptance by a stable, dependable, and protective other; (4) for confirmation of personal experience; (5) for self-definition; (6) to impact the other person; (7) for the other person to initiate; and (8) to express love.

### Relational Needs Satisfaction Scale (RNSS)

Žvelc et al. (2020) developed a scale for measuring the satisfaction of relational needs (Relational Needs Satisfaction Scale – RNSS). Contrary to the eight needs anticipated by Erskine (1998), they confirmed five relational needs in a non-clinical sample. The RNSS is composed of 20 items designed to measure five theoretical dimensions of relational needs: Support and protection, Having an Impact, Authenticity, Shared Experience, and Initiative from the Other. Items are rated on a 5-point Likert scale, ranging from 1 (*completely disagree*) to 5 (*completely agree*). Subscale scores are computed as the mean of the respective item scores, and the overall score is derived as the grand mean of all items. A higher score indicates a higher level of satisfaction with a specific dimension.

The dimension Support and Protection (Žvelc et al., 2020) is related to the experience that a person has someone in their life whom they can ask for help, protection, and support when in distress. It is related to Erskine’s need for acceptance by a stable, dependable, and protective other (Erskine et al., 1999; Erskine, 2015). Item example: “*I have a strong, stable and protective person in my life whom I can rely on*.”

The Having an Impact dimension reflects Erskine’s need to have an impact on another person (Erskine et al., 1999; Erskine, 2015). Its satisfaction is related to the experience that others accept a person’s opinion, advice, or ideas and that they can affect other people and provoke a change in them. Item example: “*Others often take my advice to heart*.”

The Shared Experience corresponds to Erskine’s need for confirmation of personal experience (Erskine et al., 1999; Erskine, 2015). Individuals with this need met have someone in their lives with similar interests, qualities and experiences. Item example: “*I know people who experience some things similarly to me.*”

The Initiative from the Other aligns with Erskine’s articulation of the need for initiative from another person (Erskine et al., 1999; Erskine, 2015). It refers to the experience of others sometimes surprising and helping us without us having to ask for it. Item example: “*Other people often help me even if I do not specifically ask them to*.”

The Authenticity dimension aligns with Erskine’s description of the needs for security, validation, and self-definition in a relationship (Erskine et al., 1999; Erskine, 2015). It encompasses feeling validated in a relationship, which in turn fosters a sense of safety and respect for one’s individuality. Item example: “*I feel free to show my feelings to others and speak my mind because I know they accept me for who I am*.”

The results of Žvelc et al.’s (2020) research did not support Erskine’s (2015) need to express love as a separate dimension. Žvelc et al. (2020) suggest that unlike other relational needs, this specific need is primarily outward-facing, as it involves a person’s active engagement with others and not merely the desire to receive love.

The original scale demonstrates adequate face validity and substantive theoretical validity, as well as satisfactory internal construct validity. Žvelc et al. (2020) identified a clear five-factor structure within the instrument. Confirmatory factor analysis indicated acceptable fits for both a five-factor correlated model and a hierarchical model, with the researchers advocating for the hierarchical model due to its theoretical coherence. This model incorporates a general factor of relational needs alongside five specific second-order dimensions, effectively capturing the covariance among the primary factors and aligning closely with the theoretical framework. Subsequent validations in Czech (Pourovȧ et al., 2020) and Turkish (Toksoy et al., 2020) populations confirmed the utility of both models, with a preference for the hierarchical structure. In contrast, a Spanish study by Iraurgi et al. (2022) provided empirical support for these models but highlighted the superior fit of a bi-factor model. Across different linguistic versions, the scale has shown reliable performance, with the total score has reliability coefficients ranging from 0.83 to 0.90.

The RNSS demonstrates suitable convergent validity across all adaptations. Researchers (Pourovȧ et al., 2020; Toksoy et al., 2020; Žvelc et al., 2020; Iraurgi et al., 2022) found significant correlations between relational needs and self-compassion, well-being, life satisfaction, distress, empathy, trusting others, emotional awareness and different facets of attachment in expected directions. Furthermore, the Czech study (Pourovȧ et al., 2020) established the scale’s measurement invariance across gender and age groups. While most validations were conducted with non-clinical samples, the Spanish study (Iraurgi et al., 2022) included both a clinical and a non-clinical group. Except for Initiative from the Other and Having an Impact, the clinical sample reported significantly lower satisfaction of relational needs, underscoring the RNSS’s discriminant validity.

### Relational needs in the work environment

Erskine (2015) describes relational needs as universal – present across all relationships and throughout the entire life cycle. The model of relational needs and the RNSS have previously been applied only in clinical and social psychology, but they may also be highly relevant in the work environment, as a growing body of research underscores the significance of social factors in the workplace (e.g., Cacioppo et al., 2003; Cohen, 2004; Lindblom et al., 2006; Diener and Ryan, 2009; Ren et al., 2018; Howard et al., 2020). Simultaneously, high quality interpersonal relationships have been shown to contribute to various organizational outcomes and employee functioning (e.g., Cacioppo et al., 2003; Cohen, 2004; Diener and Ryan, 2009), which can contribute to a number of positive effects (e.g., Dutton and Ragins, 2007; Mastroianni and Storberg-Walker, 2014; Rosales, 2016) and act as a preventive against several negative outcomes (e.g., Cohen and Wills, 1985; Persoff and Siegel, 1998; Xerri et al., 2015; Henry et al., 2018). Coworker relationships, defined as equal relationships between employees at similar status or hierarchical level within the organization (Parkes, 2003; Sias, 2009), represent the most frequent employee contact (Comer, 1991) and can often surpass time spent with family and friends in duration (Sias, 2009). The perceived social support from coworkers leads to reciprocity, reflected in higher job satisfaction and belonging to the work organization (Ferguson et al., 2012; Zhang et al., 2015). Quality relationships among colleagues are instrumental in effectively managing stress and anxiety, enhancing psychological resilience, and improve overall well-being (e.g., Persoff and Siegel, 1998; Laschinger et al., 2001; Sloan, 2012). Conversely, a lack of support from supervisors and colleagues can exacerbate stress (Blair and Littlewood, 1995; Sveinsdóttir et al., 2006; Labrique et al., 2018).

Erskine’s (2015) model of relational needs, originally developed for the clinical field, and the related Relational Needs Satisfaction Scale offer valuable insights into the interpersonal needs employees experience in their work relationships. The model of relational needs (Erskine, 1998, 2015; Žvelc et al., 2020) bears resemblance to self-determination theory (Ryan and Deci, 2000), which has been applied to the work environment (Van den Broeck et al., 2016). This theory posits that individuals will realize their full potential and optimal functioning if they satisfy three basic needs: relatedness, competence, and autonomy (Ryan and Deci, 2000). Studies investigating the link with various work-life variables have underscored the significance of the need for relatedness in the workplace, demonstrating that its satisfaction can enhance employees’ work engagement (Gorman, 2003; Trépanier et al., 2013), well-being (e.g., Patrick et al., 2007; Boezeman and Ellemers, 2009; Milyavskaya and Koestner, 2011), and job satisfaction (Van den Broeck et al., 2010).

Žvelc et al. (2020) hypothesized that relational needs are related to the needs for autonomy and relatedness. In the development of the RNSS, they proposed that the need for authenticity is to some extent similar to the need for autonomy (La Guardia and Patrick, 2008), as it involves being accepted for one’s uniqueness without efforts from others to change or control them. Furthermore, Žvelc et al. (2020) highlighted how the other four relational needs provide a more detailed understanding of the need for relatedness. Congruently, we argue that the model of relational needs may provide a more nuanced understanding of the need for relatedness in coworker relationships. Therefore, the focus of the current research is the adaptation of the RNSS to the workplace environment.

We argue that satisfaction of relational needs of employees can significantly influence their work engagement. Schaufeli and Bakker (2001) define work engagement as a positive, satisfying, work-related state of mind characterized by vitality, commitment and engagement. This construct is embedded within the social context of the workplace. Research indicates that it is positively associated with workplace elements, known as resources, motivators or energizers, which encompass social support from colleagues and the supervisor (Salanova et al., 2000; Schaufeli et al., 2002; Demerouti et al., 2010; Ehrhardt and Ragins, 2019). Fulfilling of employees’ relational needs could thus enhance their engagement at work, leading to positive attitudes toward their jobs, improved mental health, and superior performance compared to those less engaged (Schaufeli and Bakker, 2004; Kahn, 2007).

We also posit that satisfying relational needs can significantly influence work satisfaction. According to Mihalič (2008), work satisfaction is a distinctly positive emotional state arising from an individual’s experiences and evaluations of their work environment and the entirety of their work experiences. Various authors, including Herzberg et al. (1959), Warr (1987), Spector (1997), and Mullins (2001), have consistently highlighted the importance of workplace relationships in influencing work satisfaction. From the perspective of social exchange theory (e.g., Gouldner, 1960; Blau, 1964), social support is crucial for predicting and increasing work satisfaction (Ferguson et al., 2012; Zhang et al., 2015; Gerlach, 2019; Busque-Carrier et al., 2022). Employees who perceive and receive social support from their superiors and colleagues are likely to reciprocate with higher work satisfaction and organizational loyalty. The RNSS may provide deeper insights into this construct, which has widespread implications for an individual’s life. For instance, it leads to greater organizational loyalty (Brunetto and Farr-Wharton, 2003), work satisfaction is linked to lower turnover and absenteeism (Howard et al., 2012; Yarbrough et al., 2017), and enhances job performance contributing to the achievement of organizational goals and organizational effectiveness (Gorenak and Pagon, 2006). Conversely, employee dissatisfaction manifests in reduced job performance (Lambert et al., 2005), increased burnout (Whitehead and Lindquist, 1989), lower motivation (Locke, 1976), and can result in both mental and physical health issues (Garland et al., 2009).

The concept of relational needs satisfaction may also play a crucial role in understanding burnout. A lack of social support is commonly identified as a key organizational factor contributing to burnout (e.g., Lindblom et al., 2006; Cohen et al., 2023). Employees experiencing burnout often feel undervalued by their supervisors or colleagues, leading to a loss of concern for the organization and fostering critical and distrustful attitudes toward management, colleagues and supervisors (Schaufeli and Buunk, 2002; Hersh, 2022). Unfulfilled relational needs can result in feelings of emptiness, loneliness, frustration and anger, which deplete energy and hope and engender negative beliefs about self, others and life (Erskine and Moursund, 1988; Erskine et al., 1999). These feelings may overlap significantly could with burnout symptoms, underscoring unsatisfactory workplace relationships as a significant contributing factor. Research has also shown troublesome evidence that burnout can be contagious among colleagues (Bakker et al., 2005, 2007).

The current study aims to adapt the general RNSS for coworker relationships following Žvelc et al.’s (2020) recommendation to apply the scale in studying interpersonal relationships across different psychology fields. The RNSS measures the general satisfaction of five relational needs and does not discriminate between different types of relationships. In the current research, we aim to adapt the scale specifically to measure the satisfaction of relational needs in coworker relationships. The new version of the RNSS, the Workplace Relational Needs Satisfaction Scale (W-RNSS), can be used both for research purposes regarding relational needs in the workplace and for practice-based organizational applications, such as coaching and mentoring.

Our objectives are: (1) to verify whether the 5-factor structure of relational needs, validated in adult non-clinical samples across different countries (Pourovȧ et al., 2020; Toksoy et al., 2020; Žvelc et al., 2020; Iraurgi et al., 2022), is also applicable in the workplace context; (2) to assess the external construct validity of the adapted scale by examining the relationship of relational needs with basic needs as outlined in self-determination theory, work engagement, work satisfaction and burnout. We hypothesize that overall satisfaction of relational needs in coworker relationships will be: (a) positively related with the satisfaction of the three basic needs (Ryan and Deci, 2000), especially autonomy and relatedness, (b) positively related to work engagement (e.g., Wong et al., 2010; Tran et al., 2018; Ehrhardt and Ragins, 2019), (c) positively linked to work satisfaction (e.g., Fletcher and Williams, 1996; Yavas and Bodur, 1999; Peterson et al., 2003; Raabe and Beehr, 2003; Sy et al., 2006; Othman et al., 2008; Singh, 2008; Busque-Carrier et al., 2022) and (d) negatively associated with burnout (e.g., Lindblom et al., 2006; Cohen et al., 2023).

## Materials and methods

### Sample and procedure

We conducted the study through an online survey hosted on Slovenian platform 1 ka. Initially, we outlined the study’s general aims, highlighting the voluntary nature of participation and the confidentiality of responses. To ensure participants met the study’s criteria, we included a screening question asking whether respondents work in an environment with coworkers, automatically redirecting those who did not qualify to the survey’s conclusion. The survey collected demographic information and then proceeded with specific measures, including the W-RNSS, W-BNS, UWES-9, Work Satisfaction Scale, and OLBI, all administered in Slovene. On average, completing the survey took 8 min. We employed a snowball sampling method, encouraging respondents at the survey’s end to share the link with other employed or student workers. The survey link was disseminated across various social media platforms, including Facebook and Instagram profiles, Facebook groups, the student blog Psychology of Work, and to the members of the Slovenian Psychology Students’ Association.

Of the 382 respondents, who began the online survey, 11 were excluded because they reported not having coworkers, failing to meet the study’s participation criteria. In order to ensure the reliability of the data in the online survey, we required participants to answer all of the questions. Consequently, the final sample comprised 273 participants who completed the survey in full. The sample included both employees and students working in a setting with coworkers. The demographic characteristics of the sample are detailed in Table 1.

**Table 1:** Sample characteristics (*N* = 273).

Age (years)		
Mean	36.07	
Standard deviation	11.77	
Range	18–65	
Gender		
Female	217	(79.49%)
Male	56	(20.51%)
Education		
Lower vocational education	1	(0.37%)
Secondary vocational education	3	(1.10%)
V. level	40	(14.65%)
Non-university high education	17	(6.23%)
1st Bologna cycle	98	(36.00%)
2nd Bologna cycle	81	(29.67%)
Master of science degree, specialization after completing a university education study program	21	(7.69%)
3rd Bologna cycle	12	(4.40%)
Sector		
Education	63	(23.08%)
Health care	46	(16.85%)
Services	29	(10.62%)
Sports and recreation	23	(8.42%)
Sales and trade	18	(6.59%)
Social services	12	(4.76%)
Free time	12	(4.76%)
Catering	6	(2.20%)
Finance	6	(2.20%)
Building and housing	5	(1.83%)
Other	51	(18.68%)
Duration of employment in current work organization		
Up to 6 months	36	(13.19%)
Between 6 months and 1 year	53	(19.41%)
Between 1 and 5 years	75	(27.47%)
Between 5 and 10 years	34	(12.45%)
10 years and more	75	(27.47%)
Size of work organization		
Up to 10 employees	45	(16.48%)
Between 10 and including 50 employees	59	(21.61%)
Between 50 and including 250 employees	96	(35.16%)
More than 250 employees	73	(26.74%)

For the calculation of descriptive statistics for “age,” 1 participant was eliminated, as they submitted their age as “20–50”.

### Measures

#### Demographic questionnaire

The questionnaire included questions about the respondents’ gender, age, highest level of education attained, and was followed by inquiries regarding the size, sector and duration of employment at their current organization.

#### W-RNSS (Workplace Relational Needs Satisfaction Scale)

The W-RNSS (Hanc, 2023) is an adapted version of RNSS (Žvelc et al., 2020). In the current study, the original 20 items were modified to pertain specifically to relationships with coworkers. For example, the item “*I know a capable individual who would help me if I found myself in trouble*.” was revised to “*I have a capable coworker who would help me if I found myself in trouble*.” Items’ modifications were approved by the original author of the RNSS, who verified the scale’s theoretical validity. The scale retains the same 5-point rating scale and scoring system as the original RNSS.

#### W-BNS (the Work-Related Basic Needs Satisfaction Scale)

For measuring work motivation, The Work-Related Basic Needs Satisfaction Scale – W-BNS (Van den Broeck et al., 2010) was used. It comprises 18 items that assesses three needs – autonomy, relatedness and competence – as defined by self-determination theory (Ryan and Deci, 2000). It employs a 5-point rating scale (1 = *totally disagree*, 5 = *totally agree*) with eight items scored in reverse. Research supports the scale’s three-factor structure, validity, and internal reliability, with average Cronbach’s alpha coefficients of 0.79 for Autonomy, 0.83 for Competence, and 0.76 for Relatedness (Van den Broeck et al., 2016). Additionally, the scale has been shown to be independent of socially desirable responding (Colledani et al., 2018), and exhibits criterion validity (Van den Broeck et al., 2010), construct validity, and nomological validity (Colledani et al., 2018).

The current study marks the first W-BNS adaptation to Slovene. Using CFA, a modified three-factor model was accepted, demonstrating statistically superior fit over the basic model: *χ*^2^(131) = 319.25; *p* < 0.001; *χ*^2^/*df* = 2.44; CFI = 0.90; TLI = 0.88; NNFI = 0.88; RMSEA [90% CI] = 0.07; SRMR = 0.07. This model showed a better fit as evidenced by a significant chi-square difference [Δ*χ*^2^(1) = 26.21; *p* < 0.001] and lower AIC (Δ = 30.38) and BIC (Δ = 26.77) values compared to the basic model. Model fit was further improved by allowing covariance between similar items 1 (“*I feel like I can be myself at my job*.”) and 16 (“*At work, I can talk with people about things that really matter to me.*”), based on the second highest modification index (MI = 29.46). The scale demonstrated good internal consistency with an overall Cronbach’s alpha of *α* = 0.83, and subscale alphas of *α* = 0.79 for Autonomy and *α* = 0.76 for Relatedness.

#### UWES-9 (Utrecht Work Engagement Scale – short version)

The UWES-9 (Schaufeli et al., 2006; Slovene adaptation by Tement, 2014) measures a person’s work engagement. It comprises nine items, yielding an overall score and three dimensions: Vigor, Dedication and Absorption. Items are rated on a 7-point frequency scale ranging from 0 (*never*) to 6 (*always, every day*). The scale’s overall reliability ranges between 0.89 and 0.97, averaging 0.93 (Schaufeli and Bakker, 2004). Given the high correlations between the dimensions, the one factor model’s adequacy and the overall score’s reliability, the use of the scale in a unidimensional manner is recommended. In the current study the overall score demonstrated excellent reliability (*α* = 0.93) with the subscales Vigor (*α* = 0.84), Dedication (*α* = 0.88) and Absorption (*α* = 0.83) showing good reliability.

#### Work Satisfaction Scale

The Work Satisfaction Scale (Pogačnik, 1997) is a self-report measure for obtaining an estimation of a person’s general satisfaction with their work situation. It comprises 15 statements, that cover various work aspects, with responses recorded on a 5-point scale ranging from 1 (*very unsatisfied*) to 5 (*very satisfied*). The scale not only yields insights into specific working motives but also allows for the calculation of a total score or average, which reflects global work satisfaction. Its reliability, as determined across different employee samples in Slovenia, has been documented to range from *α* = 0.78 to *α* = 0.89 (Pogačnik, 2000). The scale’s validity is further supported by its correlation with the Scale of Working Motives (Pogačnik, 1997), which indirectly measures work satisfaction. In the current study, the overall’s internal consistency was *α* = 0.85.

#### OLBI (the Oldenburg Burnout Inventory)

The OLBI (Demerouti et al., 2010) is a self-report instrument that comprises 16 items, measuring two dimensions: Exhaustion and Disengagement. The scale employs a 4-point rating scale, ranging from 1 (*strongly agree*) to 4 (*strongly disagree*). Each subscale contains four items that are positively worded and four that are negatively worded. The reliability of Exhaustion ranges between *α* = 0.74 and *α* = 0.85, whereas the reliability of Disengagement ranges between *α* = 0.73 and *α* = 0.85 (Demerouti et al., 2003; Halbesleben and Demerouti, 2005; Demerouti and Bakker, 2008; Halbesleben, 2010; Sonnentag et al., 2010; Timms et al., 2012). The scale has adequate factor, convergent and discriminative validity (Demerouti et al., 2003; Halbesleben and Demerouti, 2005; Demerouti et al., 2010). In the current study, the internal consistency was *α* = 0.85 for the overall score and *α* = 0.79 for the subscales.

#### Analysis

We analyzed the data using Microsoft Excel and RStudio (Posit team, 2023). For the W-RNSS and the Slovene translation of W-BNS, we initially performed CFA, anticipating the sample size of 273 participants to be adequate, as it exceeds the minimum sample size of 200, and 10 participants per variable (Myers et al., 2011). CFAs were conducted in RStudio using the lavaan package, employing the robust MLM [maximum likelihood method with a Satorra and Bentler (1994) scaled test statistic] for parameter estimation. Model fit was evaluated using the Chi-square test (*χ*^2^), normed chi-square statistics (*χ*^2^/*df*), comparative fit index (CFI), Tucker–Lewis index (TLI), non-normed fit index (NNFI), root-mean-square error of approximation (RMSEA) with 90% CI, standardized root-mean-square residual (SRMR), Akaike information criterion (AIC) and Bayesian information criterion (BIC). An acceptable model fit was determined by CFI and NNFI values ≥0.90 (Schermelleh-Engel et al., 2003), RMSEA ≤0.06, SRMR ≤0.08 (Hu and Bentler, 1999), TLI ≥ 0.90 (Brown, 2015) and *χ*^2^/*df* ≤ 3 (Schermelleh-Engel et al., 2003). Lower AIC and BIC values indicate a better fit (Sočan, 2021). Nested models were compared using the Chi-square test.

Additionally, we conducted an overview of the data including descriptive statistics and measures of dispersion, and assessed distribution normality using the Shapiro and Wilk (1965) test. The convergent validity of the W-RNSS was examined through correlations between its dimensions and other measures, employing the Pearson correlation coefficient (using RStudio’s functions “cor.test” and “cor”). The internal consistency of the scales was evaluated using Cronbach’s alpha (using RStudio’s function “alpha”).

## Results

### Confirmatory factor analysis (factorial structure)

We conducted CFA for four distinct models: a unidimensional model, a five correlated factors model, a hierarchical model of five factors under a general second-order factor and a bi-factor model. Their comparison using the Chi-square test and the fit indices are detailed in Table 2.

**Table 2:** CFA fit indices for the Workplace Relational Needs Satisfaction Scale (*N* = 273).

Model	*χ* ^2^	*df*	χ^2^/*df*	CFI	TLI	NNFI	RMSEA [90% CI]	SRMR	AIC	BIC
F5	405.90***	160	2.54	0.92	0.90	0.90	0.08; [0.07; 0.08]	0.05	13384.91	13565.38
FH	420.66***	165	2.55	0.92	0.90	0.90	0.08; [0.07; 0.08]	0.06	13389.67	13552.10
Bi-F	290.71***	150	1.94	0.95	0.94	0.94	0.06; [0.05; 0.07]	0.04	13289.72	13506.29
F1	960.07***	170	5.65	0.74	0.71	0.71	0.13; [0.12; 0.14]	0.08	13919.08	14063.46

F5, five-factor model; FH, hierarchical model; Bi-F, bifactor model; F1, unidimensional model. Indices of fit: *χ*^2^, Chi-square; df, degrees of freedom; CFI, comparative fit index; TLI, Tucker–Lewis index; NNFI, non-normed fit index; RMSEA, root-mean-square error of approximation; CI, confidence interval; SRMR, standardized root mean square residual; AIC, Akaike information criterion; BIC, Bayesian informational criterion. ***p < 0.001.

None of the tested models met the criteria for appropriateness based on the *χ*^2^ test results (*p* < 0.001). The one-dimensional model was found to be particularly inadequate, underperforming in most fit measures and exhibiting the highest values for the information criteria AIC and BIC. A similar inadequacy of the one-dimensional model was observed by researchers in the RNSS’s Czech adaptation (Pourovȧ et al., 2020).

The goodness of fit for both the five-factor and hierarchical models was comparable, showing adequate fit according to *χ*^2^/*df*, CFI, TLI, NNFI, RMSEA, SRMR and similar AIC and BIC values. These findings align with those from previous research (Pourovȧ et al., 2020; Toksoy et al., 2020; Žvelc et al., 2020; Iraurgi et al., 2022), suggesting that a five-dimensional model accurately reflects Erskine’s (2015) theoretical framework, with the potential inclusion of a general factor. In the original study (Žvelc et al., 2020), the Czech (Pourovȧ et al., 2020) and Turkish adaptations (Toksoy et al., 2020) of the RNSS, both models demonstrated good fit, however, the researchers favored the hierarchical model for its theoretical consistency and its ability to account for the covariance among the first-order factors.

**Figure 1:**
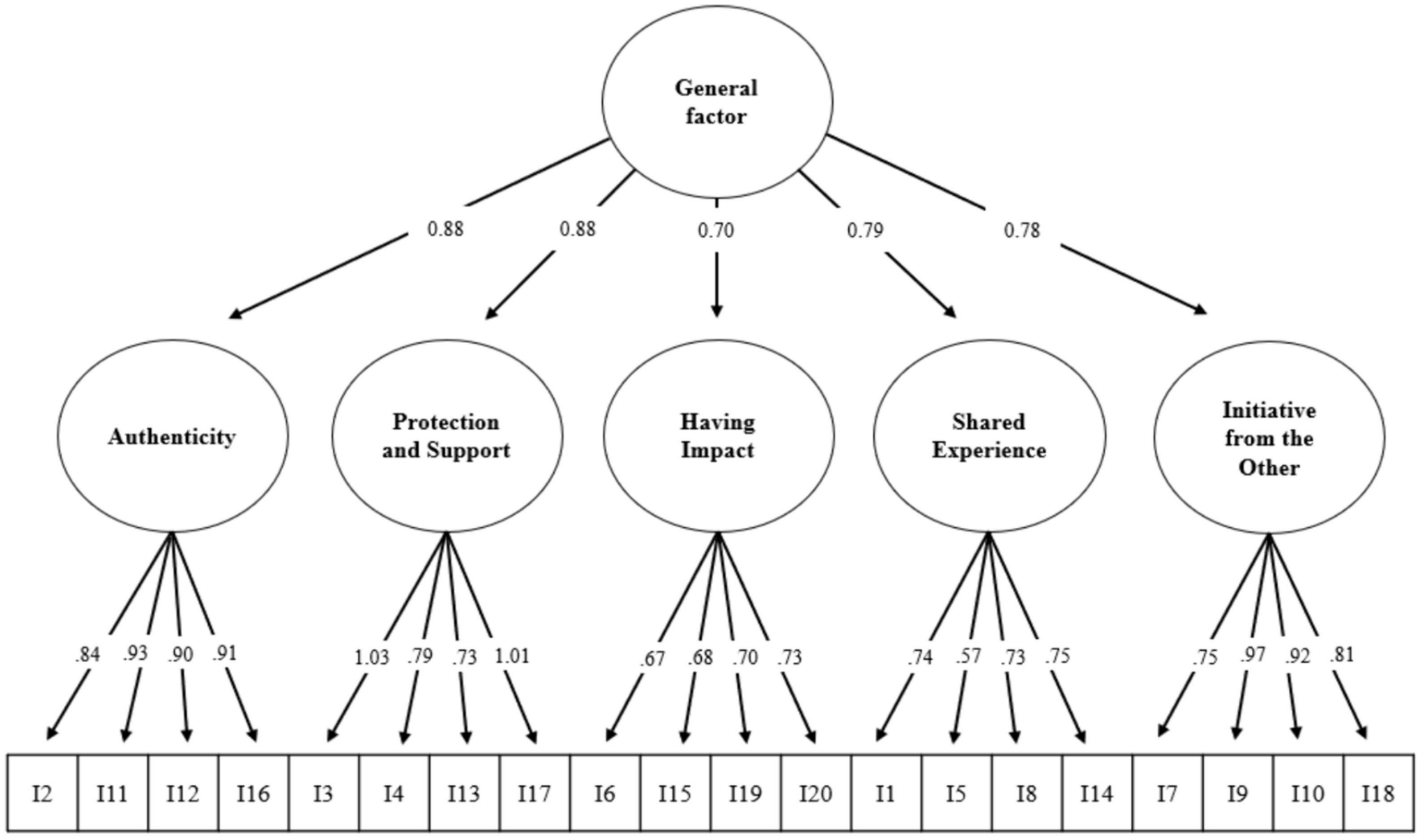
Path diagram for the hierarchical model.

**Figure 2:**
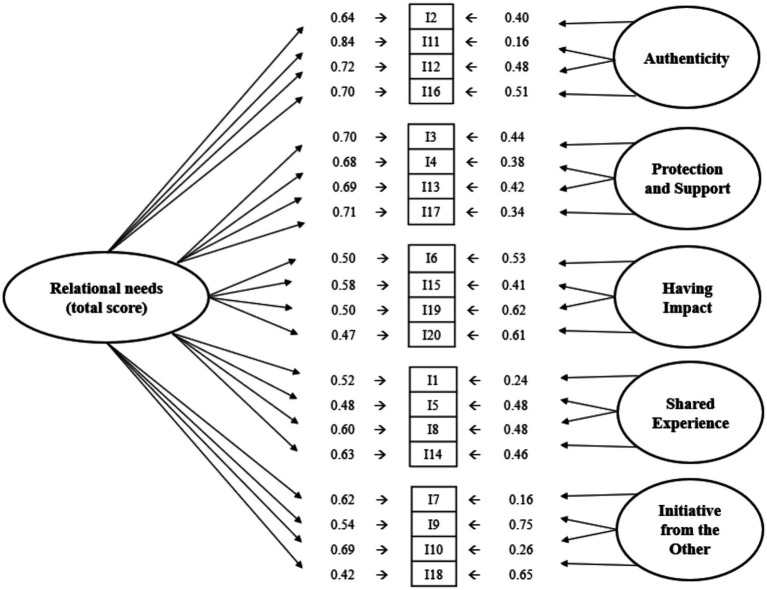
Path diagram for the bi-factor model.

Based on the fit measures (Table 2), the bi-factor model emerges as the most suitable for describing the data. It displayed consistently high values across all fit indices and recorded the lowest AIC and BIC values. Various authors (e.g., Reise et al., 2010; Chen et al., 2013) have highlighted the benefits of the bi-factor model over traditional hierarchical second-order CFA models. Its primary advantage in enabling direct assessment of how much an item or scale reflects the general factor versus specific subdimensions. The capability allows for maintaining a single latent factor while accounting for variance introduced by additional common factors. Furthermore, the bi-factor model serves as an effective baseline model for comparing fit with more restrictive models (Brown, 2015). Consequently, we employed the *χ*^2^ test to compare the nested models, specifically the hierarchical (Figure 1) and the bi-factor model (Figure 2), revealing the bi-factor model’s statistically superior fit: Δ*χ*^2^(15) = 99.11; *p* < 0.001, along with lower AIC and BIC values. This outcome lends further support to the bi-factor model as the most fitting description of the data, corroborating a similar observation made by Iraurgi et al. (2022) in RNSS’s Spanish adaptation of the scale.

Figure 2 provides a graphical representation of the bi-factor model, showcasing the factor loadings of items on both the individual dimensions and the overall score of the W-RNSS. Notably, the bi-factor model yielded higher factor loadings for the items on the overall factor, all of which were statistically significant (*p* < 0.001). This pattern mirrors findings from the Spanish validation (Iraurgi et al., 2022), with the distinction that in our study, all items have correspondingly high factor loadings on the general factor. Items 11, 12, 17, and 3 exhibited the highest loadings, whereas items 18, 20, and 5 had the lowest. Despite the emphasis on the general factor, this does not diminish the items’ contributions to the specific latent factors, which correspond to the dimensions of relational needs within coworker relationships as investigated in this study. The majority of items showed satisfactory factor loadings (>0.40) on their respective dimension (Garson, 2022). However, items 4, 17, 10 and 1 presented less desirable loadings on their specific dimensions. Items 11 and 7 were particularly notable for their low loadings on the dimensionality factors; with item 7’s loading not reaching statistical significance at the 1% risk level (*p* = 0.014).

Despite items 7 and 11 having lower saturation on their respective dimensional factors, they significantly contribute to the overall factor. Item 11, with the highest loading of *λ* = 0.84, and item 7, with a loading of *λ* = 0.62, are particularly notable for their substantial contributions to the general factor. In comparing the magnitude of factor loadings across the two types of factors, item 5 is distinguished by its uniformly low (yet still acceptably high, as per to Garson, 2022) loadings on both the overall factor and its associated dimensional factor. Conversely, item 9 demonstrates high loadings on both the general factor and its specific dimension, indicating a robust association with both the overall construct and its particular dimension.

Overall, the items demonstrating the strongest representation of their respective subscales, as indicated by the highest factor loadings, are as follows: item 16 for the Authenticity dimension, item 3 for Protection and Support, item 19 for Having Impact, items 5 and 8 for Shared Experience, and item 9 for Initiative from the Other.

We further assessed, whether relational needs at the workplace are measured in the same manner in different subsamples by multiple group factor analysis. Configural invariance is the least stringent form of invariance, indicating that the clustering of items and the factors they represent are similar across groups. This type of invariance exists if the same loadings are significant in all groups. Weak invariance (also called metric invariance) implies that the magnitude of the loadings is similar across groups. Strong invariance (also known as scalar invariance) implies that both item loadings and item intercepts are similar across groups. Strict invariance takes it a step further by requiring that residual variances are also similar across groups. To test the differences between different models, we used the differences in chi-squared (*χ*^2^), comparative fit index (CFI), and root mean square error of approximation (RMSEA). The *χ*^2^ difference is sensitive to large sample sizes (Chen, 2007), so more emphasis was placed on the difference in CFI (which should be larger than 0.010) and the difference in RMSEA (which should be larger than 0.015) to conclude that the models are different.

Since the differences in RMSEA for age did not indicate significant differences between the models, the differences in CFI were marginal, and the inspection of modification indices did not suggest any substantial improvements to the model, we concluded that measurement invariance was achieved at all levels (see Table 3).

**Table 3:** Fit indices for the invariance test of the Workplace Relational Needs Satisfaction Scale for age (*N*_below32.5_ = 136, *N*_above32.5_ = 136).

Invariance	*χ* ^2^	*df*	Δ*χ*^2^	CFI	ΔCFI	RMSEA	ΔRMSEA
Configural	446.31	300		0.952		0.060	
Weak	481.76	334	35.455	0.952	0.000***	0.057	0.003**
Strong	528.18	348	46.420***	0.941	0.011*	0.062	0.005**
Strict	569.20	354	41.018***	0.930	0.011*	0.067	0.005**

***p < 0.001, **p < 0.01, *p < 0.05.

Since both the RMSEA and CFI differences indicate invariance in the models, we concluded that measurement invariance was achieved at all levels (see Table 4).

**Table 4:** Fit indices for the invariance test of the Workplace Relational Needs Satisfaction Scale for gender (*N*_female_ = 217, *N*_male_ = 56).

Invariance	*χ* ^2^	*df*	Δ*χ*^2^	CFI	ΔCFI	RMSEA	ΔRMSEA
Configural	487.82	300		0.940		0.068	
Weak	541.97	334	54.143*	0.934	0.006**	0.068	0.000***
Strong	569.51	348	27.548*	0.929	0.004**	0.068	0.001**
Strict	607.87	354	38.359***	0.919	0.010*	0.072	0.004**

***p < 0.001, **p < 0.01, *p < 0.05.

### Descriptive statistics, reliabilities

The data analysis suggests a prevalent response style among participants, as evidenced by the predominance of answers skewed in a positive direction. Specifically, Table 5 reveals a leftward asymmetric distribution in the total score and all subscales, particularly on Protection and Support. This pattern is also apparent in the responses to individual items, notably items 13 (*M* = 4.36), 4 (*M* = 4.22), 5 (*M* = 4.01), 20 (*M* = 3.82), and 18 (*M* = 3.81) which are distinguished by their high positive mean scores. The departure from a normal distribution for the overall W-RNSS score and each subscale is confirmed by statistically significant results of the Shapiro–Wilk test.

**Table 5:** Descriptive statistics, measures of dispersion and reliabilities for the Workplace Relational Needs Satisfaction Scale (*N* = 273).

	*M*	*SD*	Min	Max	Skewness	Kurtosis	*α*	*W*
Authenticity	3.46	0.95	1.00	5.00	−0.41	−0.32	0.89	0.97***
Protection and Support	3.98	0.94	1.00	5.00	−1.07	0.44	0.87	0.88***
Having an Impact	3.61	0.76	1.00	5.00	−0.60	0.56	0.83	0.96***
Shared Experience	3.58	0.79	1.00	5.00	−0.26	−0.18	0.77	0.98***
Initiative from the Other	3.38	0.96	1.00	5.00	−0.34	−0.58	0.81	0.97***
Total	3.60	0.71	1.20	4.95	−0.68	0.40	0.93	0.97***

M, arithmetic mean; SD, standard deviation; Min, minimal value; Max, maximum value; α, Cronbach’s alpha coefficient; W, Shapiro–Wilk test statistic. ***p < 0.001.

The results represented in Table 5 confirm the W-RNSS adequate internal consistency. The reliability coefficients for the individual subscales were strong, varying from *α* = 0.77 for Shared Experience to *α* = 0.89 for Authenticity. With the overall’s scale reliability being excellent, these findings support the utility of the total score as a robust measure of an individual’s satisfaction with relational needs in coworker relationships.

### Construct validity

Table 6 indicates that all subscales of the W-RNSS are significantly positively correlated with each other (*p* < 0.001), with correlation coefficients ranging from 0.38 (indicating low correlation) to 0.67 (indicating medium or moderate correlation). This aligns with theoretical expectations, as Žvelc et al. (2020) suggest that relational needs naturally overlap. Furthermore, each dimension is also positively and significantly associated with the overall satisfaction of relational needs, underscoring the theoretical framework that the dimensions represent different aspects of relational need satisfaction. Consequently, the findings confirm that overall satisfaction of relational needs in coworker relationships is related to the satisfaction of specific relational needs.

**Table 6:** Correlations between the dimensions of the Workplace Relational Needs Satisfaction Scale (*N* = 273).

	Authenticity	Protection and support	Having impact	Shared experience	Initiative from the other	Total
Authenticity	–					
Protection and support	0.67***	–				
Having impact	0.57***	0.51***	–			
Shared experience	0.58***	0.60***	0.49***	–		
Initiative from the other	0.59***	0.60***	0.38***	0.47***	–	
Total	0.86***	0.86***	0.72***	0.77***	0.78***	–

***p < 0.001.

The current study aimed to investigate the convergent validity of the W-RNSS. The correlations between the W-RNSS total score, its subscales, and other measures, as presented in Table 7, align with our expectations and affirm the scale’s adequate construct validity. Notably, both the subscales and the total score of the W-RNSS showed significant positive correlations with the total score of the UWES-9 and its three dimensions of work engagement, ranging from weak to moderate in strength.

**Table 7:** Correlations between the dimensions of the Workplace Relational Needs Satisfaction Scale, work motivation, work engagement, work satisfaction, and burnout (*N* = 273).

	Authenticity	Protection and support	Having impact	Shared experience	Initiative from the other	Total
**UWES-9**						
Vigor	0.43***	0.35***	0.29***	0.28***	0.37***	0.44***
Dedication	0.35***	0.30***	0.25***	0.29***	0.28***	0.37***
Absorption	0.28***	0.21***	0.23***	0.20***	0.22***	0.29***
Total	0.39***	0.31***	0.28***	0.28***	0.32***	0.40***
**Work Satisfaction Scale**						
Work conditions	0.32***	0.27***	0.16**	0.20**	0.26***	0.31***
Chance of promotion	0.18**	0.14*	0.10	0.08	0.21***	0.18**
Awareness of events in the company	0.24***	0.25***	0.18**	0.21***	0.28***	0.29***
Payment and other material benefits	0.11	0.02	0.12	0.01	0.07	0.08
Coworker relationships	0.68***	0.57***	0.40***	0.46***	0.60***	0.69***
Stability of employment	0.08	0.11	0.19**	0.04	0.11	0.13*
Possibilities for professional development	0.26***	0.27***	0.23***	0.16**	0.21***	0.28***
Freedom and independence at work	0.28***	0.27***	0.24***	0.17**	0.14*	0.28***
Reputation of work	0.10	0.13*	0.11	0.13*	0.07	0.13*
Codecision at work and business	0.33***	0.32***	0.38***	0.21***	0.19**	0.36***
Creativity of work	0.33***	0.29***	0.25***	0.29***	0.22***	0.34***
Safety of work	0.19**	0.18**	0.21***	0.10	0.17**	0.21***
Immediate superior	0.33***	0.32***	0.21***	0.17**	0.25***	0.32***
Difficulty of work (physical and mental)	0.23***	0.13*	0.11	0.15*	0.11	0.19**
Interestedness of work	0.31***	0.25***	0.17**	0.23***	0.19**	0.29***
Total	0.45***	0.40***	0.35***	0.30***	0.35***	0.47***
**W-BNS**						
Autonomy	0.57***	0.45***	0.35***	0.28***	0.30***	0.49***
Competence	0.23***	0.12	0.28***	0.11	0.11	0.21***
Relatedness	0.68***	0.63***	0.48***	0.56***	0.64***	0.76***
**OLBI**						
Disengagement	−0.34***	−0.27***	−0.22***	−0.21***	−0.21***	−0.32***
Exhaustion	−0.35***	−0.23***	−0.23***	−0.09	−0.22***	−0.28***
Total	−0.39***	−0.29***	−0.26***	−0.17*	−0.25***	−0.34***

UWES-9, Utrecht Work Engagement Scale – short version; W-BNS, Workplace Basic Needs Satisfaction Scale; OLBI, Oldenburg Burnout Inventory. ***p < 0.001, **p < 0.01, *p < 0.05.

The correlations between the subscales and the total score of W-RNSS with the overall score of the Work Satisfaction Scale were also positive and significant, aligning with our expectations. Notably, the item *“relationships with colleagues*” from the Work Satisfaction Scale showed the highest correlation with the overall score of W-RNSS (*r* = 0.69). Accordingly, this item also demonstrated particularly strong correlations with the W-RNSS subscales, especially Initiative from the Other (*r* = 0.60) and Authenticity (*r* = 0.68). Conversely, the W-RNSS showed the lowest and insignificant correlations with the item *“salary and other material benefits,”* providing evidence of its discriminant validity.

The overall score of the W-RNSS was significantly positively correlated with all subscales of the W-BNS, reflecting needs as defined by self-determination theory. Predominantly, subscales of the W-RNSS were only significantly positively correlated with the Relatedness and Autonomy subscales of the W-BNS, aligning with the theoretical similarities between these models of needs as discussed by Žvelc et al. (2020). Notable exceptions were Authenticity and Having Influence subscales of the W-RNSS, which also showed significant positive correlations with the Competence scale of the W-BNS. This pattern indicates that, among all the measures used, the W-RNSS dimensions were most strongly connected to the need for relatedness within the W-BNS framework. Furthermore, the correlation between the overall W-RNSS score and Relatedness subscale of the W-BNS was the most pronounced (*r* = 0.76), underscoring the significant association between overall relational need satisfaction in coworker relationships and the need for relatedness.

As expected, both the individual subscales and the overall W-RNSS score were found to be weakly, yet significantly, negatively correlated with burnout, as indicated by both overall OLBI score and its specific dimensions. The only exception to this pattern was the correlation between the Exhaustion dimension of the OLBI and the Shared Experience subscale of the W-RNSS, which was not statistically significant.

To test the incremental validity of the W-RNSS over the W-BNS, we used a two-step hierarchical regression, as shown in Table 8. In the first step, we included the W-BNS as the predictor. In the second step, we added the W-RNSS to assess its incremental contribution.

**Table 8:** Results (unstandardized coefficients) of the two-step hierarchical regression (*N* = 273).

	Work satisfaction scale total	UWES-9 vigor	UWES-9 dedication	UWES-9 absorption	OLBI disengagement	OLBI exhaustion
**Model 1**						
*W-BNS*						
Autonomy	0.363	0.134	0.416***	0.231*	−0.127*	−0.055
Competence	0.464	0.327***	0.424***	0.597***	−0.207***	−0.154**
Relatedness	0.439	0.000	0.041	−0.055	0.000	0.024
*R* ^2^	0.045	0.061	0.162***	0.168***	0.087***	0.032***
**Model 2**						
*W-BNS*						
Autonomy	0.253	0.082	0.389***	0.200	−0.105	−0.079
Competence	0.360	0.304***	0.401***	0.577***	−0.203***	−0.154*
Relatedness	0.048	−0.125	−0,073	−0.132	0.039	0.029
*W-RNSS*						
Authenticity	0.667***	0.270***	0.193*	0.181*	−0.072	0.101*
Protection and support	0.386	0.086	0.122	0.052	0.001	−0.064
Having impact	0.299	0.022	−0.006	0.023	0.023	−0.017
Shared experience	−0.166	−0.004	0.064	0.013	−0.065	−0.009
Initiative from the other	0.254	0.089	−0.072	−0.010	−0.006	0.039
*R* ^2^	0.247***	0.239***	0.259***	0.229***	0.126***	0.054***
Δ*R*^2^	0.202***	0.178***	0.097***	0.061***	0.039*	0.022

UWES-9, Utrecht Work Engagement Scale – short version; OLBI, Oldenburg Burnout Inventory; W-BNS, Workplace Basic Needs Satisfaction Scale; W-RNSS, Workplace Relational Needs Satisfaction Scale; ***p < 0.001, **p < 0.01, *p < 0.05.

The addition of the W-RNSS significantly increased the explained variance for all outcomes except OLBI Exhaustion, as indicated by the change in *R*^2^ (Δ*R*^2^). These substantial incremental increases demonstrate the added predictive power of the W-RNSS dimensions.

## Discussion

The study aimed to develop a scale for measuring satisfaction of relational needs within coworker relationships, examining its factor structure in the process. In doing so, we drew on the original RNSS (Žvelc et al., 2020) and its adaptations across various countries (Pourovȧ et al., 2020; Toksoy et al., 2020; Iraurgi et al., 2022).

Consistent with earlier validations of the RNSS, our study confirmed the five-factor structure of the construct of relational needs. Although both the five-factor model and the hierarchical models, previously advocated in the literature (Pourovȧ et al., 2020; Toksoy et al., 2020; Žvelc et al., 2020), demonstrated adequate fit, the bi-factor model emerged as the most fitting our data. This finding aligns with the Spanish adaptation of the RNSS (Iraurgi et al., 2022), marking the first instance where the bi-factor model was evaluated. Our results suggest that responses to the scale items reflect both distinctive relational needs and an overall satisfaction of relational needs, represented by a general factor. The bi-factor model’s relevance is underscored by the total score’s excellent reliability, validating its use as a comprehensive measure of relational needs satisfaction in coworker relationships. The presence of a general factor is further evidenced by significant correlations between the W-RNSS’s total score and its subscales, supporting the theoretical premise of Erskine’s (2015) model, that the subscales encompass different dimensions of a unified construct of relational needs. Assessing measurement invariance across different age groups and genders showed that measurement invariance was achieved. This means that the measurement model operates equivalently for males and females, as well as across various age groups. In other words, the construct being measured is interpreted in the same way regardless of the respondent’s gender or age group.

The item measurement characteristics reveal a participant tendency toward reporting higher satisfaction of relational needs, a trend of negative asymmetry also observed in the Spanish (Iraurgi et al., 2022) and Czech (Pourovȧ et al., 2020) adaptations of the RNSS. Given their statistical significance and meaningful factor loadings, all items significantly contribute to the description of the general factor of relational needs within coworker relationships. Comparable to the findings from previous RNSS research (Pourovȧ et al., 2020; Toksoy et al., 2020; Žvelc et al., 2020), the bi-factor model demonstrated that items, while displaying generally lower loadings, still maintained notably high loadings on the dimensional factors. In light of this outcomes, it appears justifiable to preserve the individual dimensions or subscales of the W-RNSS.

The findings indicate the new scale possesses adequate internal consistency, with the overall score’s reliability being excellent—surpassing all the language versions of the RNSS previously developed. The subscales also demonstrated good reliability, comparable to those of the RNSS versions, as evidenced by similarly high Cronbach’s alpha values. Furthermore, the results confirm all four expectations regarding the convergent validity of the scale, which extends the theory of relational needs to the work environment.

The research confirms Žvelc et al.’s (2021) theoretical expectations regarding the relationship between relational needs satisfaction and the three basic needs defined by self-determination theory (Ryan and Deci, 2000). Overall satisfaction of relational needs in coworker relationships was found to be positively associated with the fulfillment of all three basic needs. Notable, the need for autonomy showed the strongest association with the satisfaction of the relational need for authenticity. This is in line with theoretical expectations as both concepts underscore the individual’s need for their uniqueness to be accepted by others without attempting to control or change them (Žvelc et al., 2020). The associations with the need for relatedness were particularly strong, the highest among all measured correlations. This finding underscores Žvelc et al.'s (2020) hypothesis about the critical role of relational needs in deepening our understanding of relatedness. The results suggest that individual satisfaction with communication and mutual understanding in the workplace is connected to the satisfaction of relational needs with colleagues. Moreover, the more modest correlations with the W-BNS subscale, measuring the need for competence, affirm the W-RNSS’s discriminant validity. This indicates that the scale is finely tuned to measure aspects more closely related to the quality of interpersonal relationships.

The study adds to existing research on the importance of relational factors in work engagement, job satisfaction and burnout. The findings reveal significant positive associations between W-RNSS and various facets of work engagement, aligning with prior research that demonstrates a positive link between work engagement and social support from coworkers (Salanova et al., 2000, 2003; Demerouti et al., 2001; Schaufeli and Buunk, 2002). These results suggest that the construct of relational needs offers a nuanced understanding of the interpersonal factors influencing this critical motivational aspect of work.

The findings reveal significant positive correlations between the subscales and the overall W-RNSS score and the Work Satisfaction Scale’s overall score. Notably, the highest correlations were observed with Work Satisfaction Scale’s item “*relations with colleagues*,” underscoring the W-RNSS’s convergent validity. Furthermore, the scale’s discriminant validity is supported by the statistically insignificant associations with “*pay and other material benefits,”* a factor not inherently related to the relational dimensions of work. These results affirm the critical role of social aspects in work satisfaction, especially the impact of coworker relationships, aligning with previous research (e.g., Herzberg et al., 1959; Warr, 1987; Spector, 1997; Mullins, 2001; Busque-Carrier et al., 2022). Additionally, the findings are consistent with the social exchange theory (Gouldner, 1960; Blau, 1964), which posits a reciprocal relationship between receiving social support and increased work satisfaction (Ferguson et al., 2012; Zhang et al., 2015).

The significant correlations between W-RNSS’s overall and subscale scores – apart from correlation between Exhaustion and Shared Experience – and both dimensions of burnout underscore the association between dissatisfaction of relational needs in coworker relationships and the chronic experience of stress in the workplace. These findings align with existing research highlighting the impact of interpersonal relationship quality on burnout development (Leiter and Maslach, 2004; Lindblom et al., 2006; Cohen et al., 2023). Furthermore, they corroborate Maslach’s (1998) model, which positions workplace relationships as both potential sources of emotional strain and channels for rewards, implicating them in the propagation of burnout’s adverse effects. This conceptual overlap between burnout experiences and the emotions tied to unmet relational needs (Erskine, 1998; Erskine et al., 1999) is evident. Additionally, our results support the notion of social support within the workplace as a crucial resource for managing work-related stressors (Maslach, 1998).

Furthermore, the W-RNSS scale shows high incremental validity, predicting all outcomes except exhaustion beyond what is explained by the W-BNS. This highlights the added value of the W-RNSS in capturing relational needs that significantly contribute to work engagement, satisfaction, and burnout.

Considering the significant measurement characteristics of the W-RNSS, it is evident that Erskine’s (1998) model of relational needs model is applicable to the workplace. Despite the potential for coworker relationships to be more discrete and formal (Chiaburu and Harrison, 2008), the concept of relational needs is appropriate for describing individuals’ feelings of reciprocity of response even in these relationships. Satisfaction of relational needs encompasses not just the exchange of relevant information and assistance in career development, but also the provision of instrumental and emotional support characteristic of close collegial relationships (Kram and Isabella, 1985). Accordingly, the experience of relational need satisfaction among colleagues aligns with the hallmarks of high-quality workplace relationships, such as being a source of energy, fostering connectedness and inclusiveness (Dutton and Ragins, 2007), and promoting mutual caring, respectfulness and empathy (Rosales, 2016). This study marks the first exploration of the relational needs model within a work setting, offering a promising foundation for a deeper understanding of employees’ relational needs within the work environment.

### Limitations and future research

The study’s limitations stem from sample characteristics that may affect its representativeness and, consequently, the generalizability of the findings to the broader working population. Notable, the sample contains a disproportionate number of employees from the education and healthcare sectors. Additionally, there is a significant gender imbalance, with only a fifth of the participants identifying as male. Furthermore, the absence of a question distinguishing between students and employees in the study’s questionnaire is a notable oversight. Such information would have been valuable for characterizing the sample more precisely and for exploring potential differences in relational needs satisfaction between various employment statuses.

The findings from this study pave the way for multiple research opportunities. The bi-factor model, deemed most appropriate for our data, has thus so far only been explore in the context of the RNSS’s Spanish validation (Iraurgi et al., 2022). Further studies could benefit investigating its effectiveness in other cultural settings and with additional RNSS data. The research potential of the W-RNSS extends to exploring variations in relational needs across different work modalities, employment durations in the work organization, and noted gender differences, among others. Further validation of the W-RNSS’s convergent validity is also crucial, partly through its association with various aspects of the work environment, such as organizational culture, job performance, resignation rates, absenteeism, and presentism. While the current study focuses on coworker relationships, examining hierarchical relationships, such as those between subordinates and superiors, could offer additional insights. Lastly, although the scale demonstrated suitable measurement characteristics within the specific context of a Slovenian sample validating the W-RNSS across different cultural backgrounds remains essential for confirming its universal applicability and relevance.

## Conclusion

The current study marks a significant contribution by applying Erskine’s (2015) model of relational needs to a new domain: the workplace. This extension supports the notion of relational needs’ universality (Erskine et al., 1999; Erskine, 2015), demonstrating that their satisfaction can indeed be measured within the context of workplace relationships. Previous validation studies of the RNSS (Pourovȧ et al., 2020; Toksoy et al., 2020; Žvelc et al., 2020; Iraurgi et al., 2022) have underscored the significance of meeting relational needs for mental health and well-being. Our findings further validate the relational needs model by linking it to various work-related aspects (burnout, job satisfaction, motivation, and work engagement), thereby underscoring the values of quality interpersonal relationships among coworkers.

Practically, the W-RNSS introduces valuable tools for HR departments in organizational settings to identify psychosocial risk factors among employees. By applying the scale, HR departments could gain insight into the level of satisfaction of relational needs among employees and consequently identify aspects of interpersonal relationships between coworkers, needing most enhancement. The results could thus serve as a basis for both preventive and remedial strategies aimed at enhancing work environment. The scale could also be useful for coaching and for mentoring, e.g., for identifying and improving the functioning of employees within the work environment. The scale could be included in efforts to prevent burnout, foster better communication and a positive organizational climate, enhance team cohesion, and bolster individual well-being. Hence, the W-RNSS could play a crucial role in promoting of health within the workplace.

## Data availability statement

The raw data supporting the conclusions of this article will be made available by the authors, without undue reservation.

## Ethics statement

Ethical approval was not required for the studies involving humans because we concluded that study design and the topic of research were not sensitive to require an approval of an ethics committee. The data was collected through an online survey and we concluded that highlighting the voluntary nature of participation and the confidentiality of responses was satisfactory. The studies were conducted in accordance with the local legislation and institutional requirements. The participants provided their written informed consent to participate in this study. Written informed consent was obtained from the individual(s) for the publication of any potentially identifiable images or data included in this article.

## Author contributions

VH: Conceptualization, Data curation, Formal analysis, Methodology, Validation, Writing – original draft, Writing – review & editing. GŽ: Methodology, Supervision, Writing – original draft, Writing – review & editing. BB: Supervision, Writing – original draft, Writing – review & editing.

## References

[ref1] Ainsworth M. D. S. Blehar M. C. Waters E. Wall S. (1978). Patterns of attachment: a psychological study of the strange situation. Hillsdale, NJ: Lawrence Erlbaum.

[ref2] Bakker A. B. Le Blanc P. M. Schaufeli W. B. (2005). Burnout contagion among intensive care nurses. J. Adv. Nurs. 51, 276–287. doi: 10.1111/j.1365-2648.2005.03494.x, PMID: 16033595

[ref3] Bakker A. B. Westman M. Schaufeli W. (2007). Crossover of burnout: an experimental design. Eur. J. Work Organ. Psy. 16, 220–239. doi: 10.1080/13594320701218288

[ref4] Berne E. (1961). Transactional analysis in psychotherapy: a systematic individual and social psychiatry. New York, NY: Grove Press.

[ref5] Blair A. Littlewood M. (1995). Sources of stress. J. Commun. Nurs. 9, 38–40.

[ref6] Blau P. (1964). Exchange and power in social life: Wiley.

[ref7] Boezeman E. J. Ellemers N. (2009). Intrinsic need satisfaction and the job attitudes of volunteers versus employees working in a charitable volunteer organization. J. Occup. Organ. Psychol. 82, 897–914. doi: 10.1348/096317908X383742

[ref8] Bowlby J. (1969). Attachment and loss, 1: Attachment. London: Attachment and Loss. Basic Books.

[ref9] Brown T. A. (2015). Confirmatory factor analysis for applied research. 2nd Edn. New York, NY: The Guilford Press.

[ref10] Brunetto Y. Farr-Wharton R. (2003). Using social identity theory to explain the job satisfaction of public sector employees. Int. J. Public Sect. Manag. 15, 534–551. doi: 10.1108/09513550210448571

[ref11] Busque-Carrier M. Ratelle C. F. Le Corff Y. (2022). Work values and job satisfaction: the mediating role of basic psychological needs at work. J. Career Dev. 49, 089484532110438–089484532111401. doi: 10.1177/08948453211043878

[ref12] Cacioppo J. T. Hawkley L. C. Berntson G. G. (2003). The anatomy of loneliness. Curr. Dir. Psychol. Sci. 12, 71–74. doi: 10.1111/1467-8721.01232

[ref13] Chen F. F. (2007). Sensitivity of goodness of fit indexes to lack of measurement invariance. Struct. Equ. Model. 14, 464–504. doi: 10.1080/10705510701301834

[ref14] Chen F. F. Jing Y. Hayes A. Lee J. M. (2013). Two concepts or two approaches? A bifactor analysis of psychological and subjective well-being. J. Happiness Stud. 14, 1033–1068. doi: 10.1007/s10902-012-9367-x

[ref15] Chiaburu D. S. Harrison D. A. (2008). Do peers make the place? Conceptual synthesis and meta-analysis of coworker effects on perceptions, attitudes, OCBs, and performance. J. Appl. Psychol. 93, 1082–1103. doi: 10.1037/0021-9010.93.5.1082, PMID: 18808227

[ref16] Cohen S. (2004). Social relationships and health. Am. Psychol. 59, 676–684. doi: 10.1037/0003-066X.59.8.676 15554821

[ref17] Cohen C. Pignata S. Bezak E. Tie M. Childs J. (2023). Workplace interventions to improve well-being and reduce burnout for nurses, physicians and allied healthcare professionals: a systematic review. BMJ Open 13:e071203. doi: 10.1136/bmjopen-2022-071203, PMID: 37385740 PMC10314589

[ref18] Cohen S. Wills T. A. (1985). Stress, social support, and the buffering hypothesis. Psychol. Bull. 98, 310–357. doi: 10.1037/0033-2909.98.2.310 3901065

[ref19] Colledani D. Capozza D. Falvo R. Di Bernardo G. A. (2018). The work-related basic need satisfaction scale: an Italian validation. Front. Psychol. 9:1859. doi: 10.3389/fpsyg.2018.01859, PMID: 30333778 PMC6176063

[ref20] Comer D. R. (1991). Organizational newcomers’ acquisition of information from peers. Manag. Commun. Q. 5, 64–89. doi: 10.1177/0893318991005001004

[ref21] Demerouti E. Bakker A. B. (2008). “The Oldenburg burnout inventory: a good alternative to measure burnout and engagement” in Handbook of Stress and Burnout in Health Care, Ed. J. Halbesleben, Hauppauge, NY: Nova Science Publishers, Inc. 65–78.

[ref9001] Demerouti E. Bakker A. B. Nachreiner F. Schaufeli W. B. (2001). The job demands-resources model of burnout. Journal of Applied Psychology, 86, 499–512. doi: 10.1037/0021-9010.86.3.499 11419809

[ref22] Demerouti E. Kantas A. Vardakou I. (2003). The convergent validity of two burnout instruments. Eur. J. Psychol. Assess. 19, 12–23. doi: 10.1027//1015-5759.19.1.12

[ref23] Demerouti E. Mostert K. Bakker A. B. (2010). Burnout and work engagement: a thorough investigation of the independency of both constructs. J. Occup. Health Psychol. 15, 209–222. doi: 10.1037/a0019408, PMID: 20604629

[ref24] Diener E. Ryan K. (2009). Subjective well-being: a general overview. S. Afr. J. Psychol. 39, 391–406. doi: 10.1177/008124630903900402

[ref25] Dutton J. E. Ragins B. R. (2007). Exploring positive relationships at work: building a theoretical and research foundatio*n*. New York, NY: Lawrence Erlbaum Associates Publishers.

[ref26] Ehrhardt K. Ragins B. R. (2019). Relational attachment at work: a complementary fit perspective on the role of relationships in organizational life. Acad. Manag. J. 62, 248–282. doi: 10.5465/amj.2016.0245

[ref27] Erskine R. G. (1998). The therapeutic relationship: integrating motivation and personality theories. Trans. Anal. J. 28, 132–141. doi: 10.1177/036215379802800206

[ref28] Erskine R. G. (2015). Relational patterns, therapeutic presence: concepts and practice of integrative psychotherapy. London: Karnac Books.

[ref29] Erskine R. G. Moursund J. P. (1988). Integrative psychotherapy in action. Newbury Park, CA: Sage Publication.

[ref30] Erskine R. G. Moursund J. P. Trautmann R. L. (1999). Beyond empathy: a therapy of contact-in-relationship. Philadelphia: Brunner/Mazel.

[ref32] Fairbairn W. R. D. (1954). An object-relations theory of the personality. New York, NY: Basic Books.

[ref33] Fairbairn W. R. D. (1986/1941). “A revised psychopathology of the psychoses and psychoneuroses” in Essential papers on object relations. ed. Buckley P.. (New York, NY: New York University Press), 71–101.

[ref34] Ferguson M. Carlson D. Zivnuska S. Whitten D. (2012). Support at work and home: the path to satisfaction through balance. J. Vocat. Behav. 80, 299–307. doi: 10.1016/j.jvb.2012.01.001

[ref35] Fletcher C. Williams R. (1996). Performance management, job satisfaction and organizational commitment. Br. J. Manag. 7, 169–179. doi: 10.1111/j.1467-8551.1996.tb00112.x

[ref36] Garland B. E. Mccarty W. P. Zhao R. (2009). Job satisfaction and organizational commitment in prisons: an examination of psychological staff, teachers, and unit management staff. Crim. Justice Behav. 36, 163–183. doi: 10.1177/0093854808327343

[ref37] Garson G. D. (2022). Factor analysis and dimension reduction in R: a social Scientist's toolkit. 1st Edn. London: Routledge.

[ref38] Gerlach G. I. (2019). Linking justice perceptions, workplace relationship quality and job performance: the differential roles of vertical and horizontal workplace relationships. German J. Hum. Res. Manag. 33, 337–362. doi: 10.1177/2397002218824320

[ref39] Gorenak I. Pagon M. (2006). Vpliv organizacijskega komuniciranja na zadovoljstvo policistov pri delu. Organizacija 39, 247–253.

[ref40] Gorman B. (2003). Employee engagement after two decades of change. Strateg. Commun. Manag. 7, 14–17.

[ref41] Gouldner A. W. (1960). The norm of reciprocity: a preliminary statement. Am. Sociol. Rev. 25, 161–178. doi: 10.2307/2092623

[ref42] Guntrip H. (1992/1968). Schizoid phenomena, object relations and the self. London: Karnac Books.

[ref43] Halbesleben J. R. (2010). “A meta-analysis of work engagement: relationships with burnout, demands, resources, and consequences” in Work engagement: a handbook of essential theory and research. eds. Bakker A. B. Leiter M. P. (New York, NY: Psychology Press), 102–117.

[ref44] Halbesleben J. R. B. Demerouti E. (2005). The construct validity of an alternative measure of burnout: investigating the English translation of the Oldenburg burnout inventory. Work Stress. 19, 208–220. doi: 10.1080/02678370500340728

[ref45] Hanc V. (2023). Relacijske potrebe v delovnem okolju: magistrsko delo [Master’s thesis, V. Hanc]. Repository of the university of Ljubljana. Available at: https://repozitorij.uni-lj.si/IzpisGradiva.php?lang=eng&id=150535

[ref46] Henry J. Eshleman J. Moniz D. R. (2018). The dysfunctional library: challenges and solutions to workplace relationships. Chicago, IL: American Library Association.

[ref47] Hersh M. A. (2022). “Relational patterns and fulfillment of needs” in The thriving therapist: sustainable self-care to prevent burnout and enhance well-being. ed. Hersh M. A. (Washington, DC: American Psychological Association), 137–143.

[ref48] Herzberg F. Mausner B. Snyderman B. B. (1959). The motivation to work. 2. izdaja Edn. New York, NY: John Wiley.

[ref49] Howard M. C. Cogswell J. E. Smith M. B. (2020). The antecedents and outcomes of workplace ostracism: a meta-analysis. J. Appl. Psychol. 105, 577–596. doi: 10.1037/apl0000453, PMID: 31556627

[ref50] Howard K. J. Howard J. T. Smyth A. F. (2012). “The problem of absenteeism and presenteeism in the workplace” in Handbook of occupational health and wellness. eds. Gatchel R. Schultz I. (New York, NY: Springer), 151–179.

[ref51] Hu L.-T. Bentler P. M. (1999). Cutoff criteria for fit indexes in covariance structure analysis: conventional criteria versus new alternatives. Struct. Equ. Model. 6, 1–55. doi: 10.1080/10705519909540118

[ref52] Iraurgi I. Gómez-Marroquín I. Erskine R. Mauriz A. Martínez-Rodríguez S. Gorbeña S.. (2022). Adaptation to Spanish of the "relational needs satisfaction scale": translation and psychometric testing. Front. Psychol. 13:992205. doi: 10.3389/fpsyg.2022.992205, PMID: 36081737 PMC9445878

[ref53] Kahn W. A. (2007). “Meaningful connections: positive relationships and attachments at work” in Exploring positive relationships at work: Building a theoretical and research foundation, Eds. J. E. Dutton and B. R. Ragins. (New York, NY: Lawrence Erlbaum Associates Publishers), 189–206.

[ref54] Kohut H. (1971). The analysis of the self: a systematic approach to the psychoanalytic treatment of narcissistic personality disorders. New York, NY: University of Chicago Press.

[ref55] Kohut H. (1977). The restoration of the self. Oxford: University of Chicago Press.

[ref56] Kohut H. (1984). Introspection, empathy, and semicircle of mental health. Emot. Behav. Monog. 3, 347–375.

[ref57] Kram K. E. Isabella L. A. (1985). Mentoring alternatives: the role of peer relationships in career development. Acad. Manag. J. 28, 110–132. doi: 10.2307/256064

[ref58] La Guardia J. G. Patrick H. (2008). Self-determination theory as a fundamental theory of close relationships. Can. Psychol. 49, 201–209. doi: 10.1037/a0012760

[ref59] Labrique A. B. Wadhwani C. Williams K. A. Lamptey P. Hesp C. Luk R.. (2018). Best practices in scaling digital health in low and middle income countries. Glob. Health 14:103. doi: 10.1186/s12992-018-0424-z, PMID: 30390686 PMC6215624

[ref60] Lambert E. G. Edwards C. Camp S. Saylor W. (2005). Here today, gone tomorrow, back again the next day: absenteeism and its antecedents among federal correctional staff. J. Crim. Just. 33, 165–175. doi: 10.1016/j.jcrimjus.2004.12.008

[ref61] Laschinger H. K. Finegan J. Shamian J. Wilk P. (2001). Impact of structural and psychological empowerment on job strain in nursing work settings: expanding Kanter’s model. J. Nurs. Adm. 31, 260–272. doi: 10.1097/00005110-200105000-00006, PMID: 11388162

[ref62] Leiter M. P. Maslach C. (2004). “Areas of worklife: a structured approach to organizational predictors of job burnout” in Emotional and physiological processes and positive intervention strategies. eds. Perrewé P. Ganster D. C. (Chicago, IL: Elsevier), 91–134.

[ref63] Lindblom K. Linton S. Lundholm C. Bryngelsson I. (2006). Burnout in the working population: relations to psychosocial work factors. Int. J. Behav. Med. 13, 51–59. doi: 10.1207/s15327558ijbm1301_7 16503841

[ref64] Locke E. A. (1976). “The nature and causes of job satisfaction” in Handbook of industrial and organizational psychology. ed. Dunnette M. D. (Oxford: Rand McNally), 1297–1343.

[ref65] Maslach C. (1998). “A multidimensional theory of burnout” in Theories of organizational stress. ed. Cooper C. L. (Ljubljana: Oxford University Press), 68–85.

[ref66] Mastroianni K. Storberg-Walker J. (2014). Do work relationships matter? Characteristics of workplace interactions that enhance or detract from employee perceptions of well-being and health behaviors. Health Psychol. Behav. Med. 2, 798–819. doi: 10.1080/21642850.2014.933343, PMID: 25750820 PMC4346030

[ref67] Mihalič R. (2008). Povečajmo zadovoljstvo in pripadnost zaposlenih: praktični nasveti, metodologija, interni akt in model upodabljanja za upravljanje in merjenje zadovoljstva in pripadnosti zaposlenih, z ukrepi za večje zadovoljstvo pri delu in pripadnost organizaciji. London: Založba Mihalič in Partner.

[ref68] Milyavskaya M. Koestner R. (2011). Psychological needs, motivation, and well-being: a test of self-determination theory across multiple domains. Personal. Individ. Differ. 50, 387–391. doi: 10.1016/j.paid.2010.10.029

[ref69] Mullins L. J. (2001). Management and organizational behavior. Ljubljana: FT Pitman.

[ref70] Myers N. D. Ahn S. Jin Y. (2011). Sample size and power estimates for a confirmatory factor analytic model in exercise and sport: a Monte Carlo approach. Res. Q. Exerc. Sport 82, 412–423. doi: 10.1080/02701367.2011.10599773, PMID: 21957699

[ref71] Othman A. K. Abdullah H. S. Ahmad J. B. (2008). Emotional intelligence, emotional labour and work effectiveness in service organizations: a proposed model. Vis. J. Bus. Perspect. 12, 31–42. doi: 10.1177/09722629080120010

[ref72] Parkes K. R. (2003). Shiftwork and environment as interactive predictors of work perceptions. J. Occup. Health Psychol. 8, 266–281. doi: 10.1037/1076-8998.8.4.266, PMID: 14570523

[ref73] Patrick H. Knee C. R. Canevello A. Lonsbary C. (2007). The role of need fulfillment in relationship functioning and well-being: a self-determination theory perspective. J. Pers. Soc. Psychol. 92, 434–457. doi: 10.1037/0022-3514.92.3.434, PMID: 17352602

[ref74] Persoff I. L. Siegel P. H. (1998). Tax professionals, peer relationships, CPA firm restructuring: a grounded theory approach. Mid Atlantic J. Bus. 34, 125–140.

[ref75] Peterson D. K. Puia G. M. Suess F. R. (2003). “Yo Tengo La Camiseta (I have the shirt on)”: an exploration of job satisfaction and commitment among workers in Mexico. J. Leadersh. Organ. Stud. 10, 73–88. doi: 10.1177/107179190301000208

[ref76] Pogačnik V. (1997). Lestvica delovne motivacije. Thousand Oaks, CA: Produktivnost, Center za psihodiagnostična sredstva.

[ref77] Pogačnik V. (2000). Uporaba Lestvice delovnega zadovoljstva v slovenskih podjetjih. Psihološka Obzorja 9, 105–114.

[ref78] Posit team (2023). RStudio: integrated development environment for R. Posit Software, PBC. Available at: http://www.posit.co/

[ref79] Pourovȧ M. Rihȧcek T. Žvelc G. (2020). Validation of the Czech version of the relational needs satisfaction scale. Front. Psychol. 11:359. doi: 10.3389/fpsyg.2020.00359, PMID: 32210881 PMC7067918

[ref80] Raabe B. Beehr T. A. (2003). Formal mentoring versus supervisor and coworker relationships: differences in perceptions and impact. J. Organ. Behav. 24, 271–293. doi: 10.1002/job.193

[ref81] Reise S. P. Moore T. M. Haviland M. G. (2010). Bifactor models and rotations: exploring the extent to which multidimensional data yield univocal scale scores. J. Pers. Assess. 92, 544–559. doi: 10.1080/00223891.2010.496477, PMID: 20954056 PMC2981404

[ref82] Ren D. Wesselmann E. D. Williams K. D. (2018). Hurt people hurt people: ostracism and aggression. Curr. Opin. Psychol. 19, 34–38. doi: 10.1016/j.copsyc.2017.03.026, PMID: 29279219

[ref83] Rosales R. (2016). Energizing social interactions at work: an exploration of relationships that generate employee and organizational thriving. Open J. Soc. Sci. 4, 29–33. doi: 10.4236/jss.2016.49004

[ref84] Ryan R. M. Deci E. L. (2000). Self-determination theory and the facilitation of intrinsic motivation, social development, and well-being. Am. Psychol. 55, 68–78. doi: 10.1037/0003-066X.55.1.68, PMID: 11392867

[ref85] Salanova M. Llorens S. Cifre E. Martínez I. M. Schaufeli W. B. (2003). Perceived collective efficacy, subjective well-being, and task performance among electronic work groups: an experimental study. Small Group Res. 34, 43–73. doi: 10.1177/1046496402239577

[ref86] Salanova M. Schaufeli W. B. Llorens Gumbau S. Silla P. Grau Gumbau R. M. (2000). Desde el burnout al engagement: Â¿ una nueva perspectiva? J. Work Organ. Psychol. 16, 117–134.

[ref87] Satorra A. Bentler P. M. (1994). Corrections to test statistics and standard errors in covariance structure analysis. In Eye A. von Clogg C. C. (Eds.), Latent variables analysis: applications for developmental research (399–419). The Atrium, Southern Gate, Chichester: Sage.

[ref88] Schaufeli W. Bakker A. B. (2001). Work and well-being: towards a positive occupational health psychology. Gedrag Organisatie 14, 229–253.

[ref89] Schaufeli W. B. Bakker A. B. (2004). UWES Utrecht work engagement scale preliminary manual. Occupational Health Psychology Unit, Utrecht University. Available at: https://www.wilmarschaufeli.nl/downloads/

[ref90] Schaufeli W. B. Bakker A. B. Salanova M. (2006). The measurement of work engagement with a short questionnaire: a cross-national study. Educ. Psychol. Meas. 66, 701–716. doi: 10.1177/0013164405282471

[ref91] Schaufeli W. B. Buunk B. P. (2002). “Burnout: an overview of 25 years of research and theorizing” in The handbook of work and Health Psychology. eds. Schabracq M. J. Winnubst J. A. M. Cooper C. L. (Thousand Oaks, CA: John Wiley & Sons), 383–425.

[ref9002] Schaufeli W. B. Salanova M. González-Romá V. (2002). The measurement of engagement and burnout: A two sample confirmatory factor analytic approach. Journal of Happiness Studies, 3, 71–92. doi: 10.1023/A:1015630930326

[ref92] Schermelleh-Engel K. Moosbrugger H. Muller H. (2003). Evaluating the fit of structural equation models: tests of significance and descriptive goodness-of-fit measures. Methods Psychol. Res. 8, 23–74.

[ref93] Sedlar N. (2015). Razvoj postopka vrednotenja dejavnikov tveganja, povezanih z negativnimi izidi poklicnega stresa: doktorska disertacija. Ljubljana: Oddelek za Psihologijo, Filozofska Fakulteta.

[ref94] Shapiro S. S. Wilk M. B. (1965). An analysis of variance test for normality (complete samples). Biometrika 52, 591–611. doi: 10.1093/biomet/52.3-4.591

[ref95] Sias P. M. (2009). Organizing relationships: traditional and emerging perspectives on workplace relationships. Thousand Oaks, CA: SAGE Publications, Inc.

[ref96] Singh K. (2008). Emotional intelligence & work place effectiveness. Indian J. Ind. Relat. 44, 292–302.

[ref97] Sloan M. M. (2012). Unfair treatment in the workplace and worker well-being: the role of coworker support in a service work environment. Work. Occup. 39, 3–34. doi: 10.1177/0730888411406555

[ref98] Sočan G. (2021). Psihometrična analiza s programom R. Thousand Oaks, CA: Znanstvena Založba Filozofske Fakultete.

[ref99] Sonnentag S. Volmer J. Spychala A. (2010). Job performance, vol. 1: Thousand Oaks, CA: Sage, 427–447.

[ref100] Spector P. E. (1997). Job satisfaction: application, assessment, causes and consequences. Thousand Oaks, CA: SAGE.

[ref101] Stern D. (1985). The interpersonal world of the infant: a view from psychoanalysis and Developmental Psychology. New York, NY: Basic Books.

[ref102] Sveinsdóttir H. Biering P. Ramel A. (2006). Occupational stress, job satisfaction, and working environment among Icelandic nurses: a cross-sectional questionnaire survey. Int. J. Nurs. Stud. 43, 875–889. doi: 10.1016/j.ijnurstu.2005.11.002, PMID: 16360157

[ref103] Sy T. Tram S. O’Hara L. A. (2006). Relation of employee and manager emotional intelligence to job satisfaction and performance. J. Vocat. Behav. 68, 461–473. doi: 10.1016/j.jvb.2005.10.003

[ref104] Tement S. (2014). The role of personal and key resources in the family-to-work enrichment process. Scand. J. Psychol. 55, 489–496. doi: 10.1111/sjop.12146, PMID: 25040786

[ref105] Timms C. Brough P. Graham D. (2012). Burnt-out but engaged: the co-existence of psychological burnout and engagement. J. Educ. Adm. 50, 327–345. doi: 10.1108/09578231211223338

[ref106] Toksoy Ş. Cerit C. Aker A. Žvelc G. (2020). Relational needs satisfaction scale: reliability and validity study in Turkish. Anatolian J. Psychiatry 21:1. doi: 10.5455/apd.115143

[ref107] Tran K. T. Nguyen P. V. Dang T. T. U. Ton T. N. B. (2018). The impacts of the high-quality workplace relationships on job performance: a perspective on staff nurses in Vietnam. Behav. Sci. 8:109. doi: 10.3390/bs8120109, PMID: 30477199 PMC6316783

[ref108] Trépanier S. G. Fernet C. Austin S. (2013). Workplace bullying and psychological health at work: the mediating role of satisfaction of needs for autonomy, competence and relatedness. Work Stress 27, 123–140. doi: 10.1080/02678373.2013.782158

[ref109] Van den Broeck A. Ferris D. L. Chang C.-H. Rosen C. C. (2016). A review of self-determination theory’s basic psychological needs at work. J. Manag. 42, 1195–1229. doi: 10.1177/0149206316632058

[ref110] Van den Broeck A. Vansteenkiste M. De Witte H. Soenens B. Lens W. (2010). Capturing autonomy, competence, and relatedness at work: construction and initial validation of the work-related basic need satisfaction scale. J. Occup. Organ. Psychol. 83, 981–1002. doi: 10.1348/096317909X481382

[ref111] Warr P. (1987). Work, unemployment, and mental health. Oxford: Oxford University Press.

[ref112] Whitehead J. T. Lindquist C. A. (1989). Determinants of correctional officers' professional orientation. Justice Q. 6, 69–87. doi: 10.1080/07418828900090051

[ref113] Winnicot D. W. (1986/1960). “The theory of the parent-infant relationship” in Essential papers on object relations. ed. Buckley P. (New York, NY: New York University Press), 233–254.

[ref9003] Wong C. A. Spence Laschinger H. K. Cummings G. G. (2010). Authentic leadership and nurses’ voice behaviour and perceptions of care quality. Journal of Nursing Management, 18, 889–900. doi: 10.1111/j.1365-2834.2010.01113.x 21073563

[ref114] Xerri M. J. Nelson S. Brunetto Y. (2015). Importance of workplace relationships and attitudes toward organizational change in engineering asset-management organizations. J. Manag. Eng. 31, 04014074. doi: 10.1061/(ASCE)ME.1943-5479.0000306

[ref115] Yarbrough S. Martin P. Alfred D. McNeill C. (2017). Professional values, job satisfaction, career development, and intent to stay. Nurs. Ethics 24, 675–685. doi: 10.1177/0969733015623098, PMID: 26811397

[ref116] Yavas U. Bodur M. (1999). Satisfaction among expatriate managers: correlates and consequences. Career Dev. Int. 4, 261–269. doi: 10.1108/13620439910279743

[ref117] Zhang L. Lin Y. Wan F. (2015). Social support and job satisfaction: elaborating the mediating role of work-family interface. Curr. Psychol. 34, 781–790. doi: 10.1007/s12144-014-9290-x

[ref118] Žvelc G. Jovanoska K. Žvelc M. (2020). Development and validation of the relational needs satisfaction scale. Front. Psychol. 11:901. doi: 10.3389/fpsyg.2020.00901, PMID: 32587540 PMC7298105

